# Basal Cell Carcinoma of the Head and Neck Region: A Retrospective Analysis of Completely Excised 331 Cases

**DOI:** 10.1155/2014/858636

**Published:** 2014-04-17

**Authors:** Duriye Deniz Demirseren, Candemir Ceran, Berrak Aksam, Mustafa Erol Demirseren, Ahmet Metin

**Affiliations:** ^1^Department of Dermatology, Ataturk Training and Research Hospital, Bilkent Way, 06800 Ankara, Turkey; ^2^Department of Plastic Reconstructive and Aesthetic Surgery, Ataturk Training and Research Hospital, 06800 Ankara, Turkey

## Abstract

The aim of the study is to analyze all completely excised BCCs in the head and neck region with regard to age, sex, personal and familial history, skin type, tumor localization and size, histopathological subtype of tumor, reconstruction method, and recurrence rates. Incompletely excised BCCs were not included in this study since incomplete excision is the most important preventable risk factor for recurrence. In 320 patients, 331 lesions were retrospectively evaluated by dividing into the following 8 subunits: scalp, frontotemporal, orbital, nose, cheek, auricula, perioral, and chin-neck area. Most of the patients were in 60–70 age group (34.7%). The nose (32.3%) was the most common site of presentation. Clinically, all lesions and, histopathologically, most of the lesions (42.2%) presented were of the nodular type. All cases of recurrence after complete excision (*n* = 9, 2.7%) were located in the median parts of the head and neck region and were mainly diagnosed histopathologically as sclerotic and micronodular. Even though completely excised, head and neck region BCCs, especially which are more prone to recurrence due to anatomical and histopathological properties, should be more closely monitored in order to decrease morbidity and health care costs.

## 1. Introduction 

Basal cell carcinoma (BCC) is the most common type of skin cancer in the head and neck region. The incidence of BCC is on the rise, and it represents approximately 65% of all skin carcinomas [1–3]. BCC is not usually life threatening, unlike malignant melanoma, but it is locally invasive and may lead to considerable morbidity and complications [4]. BCC is the most frequent tumor type among the US population and also has a strongly rising incidence in Europe. Although, the incidence of BCC in Turkey is not accurately known, we think that there is a rise in the number of patients who are admitted to medical facilities [2]. With the increase in the incidence of BCC, even though mortality is relatively low, the morbidity and treatment-related costs represent a significant burden to health care systems. Treatment options include medical and surgical modalities. The first therapy of choice is generally surgical excision, with safe surgical margins. Recurrence is more common, especially with positive peripheral margins and certain types of BCC, like morpheaform forms. Recurrent BCC tends to be biologically more aggressive than primary lesions [1]. Positive surgical margins in primary BCC excisions in the head and neck region were reported to be 3–20% in the literature [5]. Recurrence rates in incompletely excised patients were also reported at 26–67% [6].

In our study, we present a case with a series of 320 patients with 331 BCCs, involving the head and neck region, and who were treated with complete surgical excision. The aim of this paper is to describe the clinical, histopathological, and epidemiological features of completely excised BCC and also to evaluate clinical features of the recurrent cases with previous histologically clear margins in the head and neck region.

## 2. Material and Methods

We performed a retrospective review of the database of our institution, Ataturk Training and Research Hospital, which has a wide acceptance of patients from the capital city Ankara and surrounding cities. All patients between January 2008 and January 2013 with head and neck BCCs were evaluated. Only completely excised primary cases with histopathologically confirmed BCCs were included in this study. Patients who were lost to follow-up before one year were not included.

All 331 lesions of 320 patients were categorized with regard to age, sex, personal and family history, skin type, tumor location, size, clinical and histopathological subtypes, reconstruction method, and recurrence rates. In order to achieve a systematic analysis of the collected data, the head and neck region were divided into the following 8 subunits: scalp, frontotemporal, orbital, nose, cheek, auricula, perioral, and chin-neck area. Lesions of each subunit were evaluated according to previously mentioned variables. The diagnosis of BCC was clinically undertaken first, but, in some cases when the clinical diagnose was not clear, the dermatologist performed a pretreatment biopsy. Most of the operations were performed under local anesthesia. Excision margins were planned, depending on the clinical features, size, and location of the lesion. With loupe (×5) magnification, the excision margin was marked in the dermatology department. While the 3 mm safe margin excision was used in small and well-defined lesions, where up to 10 mm safe margins were chosen for large tumors and unclear borders, deep margins were systematically located in the subcutaneous tissue or were more deeply situated, according to the tissue characteristics of the region. Incomplete excision was defined as a residual tumor at, or within, 1 mm of the lateral or deep incision margins. All incompletely excised lesions, whether or not they were reexcised, were excluded from the study. All excision materials underwent routine histopathological examination by the pathology department and were all confirmed as BCC. Methods of reconstruction were primary closure, skin grafts, and local and distant flaps. All patients were documented, and preoperative and postoperative photographs were taken. Routine outpatient controls were planned as postoperative one week, one month, three months, six months, and one year. Afterwards, the patients were called in for yearly controls. In some cases, extra control visits were added due to recurrence or a patient's need.

Statistical analysis was performed using SPSS 16.0 software (Chicago, IL, USA). Nonnormally distributed continuous variables were expressed as median, and categorical variables were expressed as numbers and percentages. Mann-Whitney* U* test was used in comparison with the continuous variables, while the chi-square test was used in comparison with the categorical variables. The level of statistical significance was considered as *P* < 0.05.

## 3. Results

A sample of 320 patients, presenting 331 histopathologically confirmed BCC of the head and neck, were identified in the retrospective review. There were 176 (55%) men and 144 (45%) women. In the whole head and neck region, there was no statistically significant difference between the male and female population. When the subunits were evaluated, scalp, frontotemporal, and auricular regions presented male predominance, while the perioral region presented female predominance (*P* < 0.05). The mean age of the patients was 67.3 ± 12.76. Tumors were rare below the age of 20, with only one case in the present study who revealed no history of an underlying disease such as nevoid BCC syndrome or xeroderma pigmentosum. Higher prevalence of patients (34.7%) was noted in the 60–70 age group which was found to be statistically significant (*P* < 0.05). There was no statistically meaningful difference noted among the subunits.

The personal history of chronic sun exposure which is defined by spending every working day in open areas due to occupational reasons was reported in 199 (62.2%) cases. One hundred and thirty (40.6%) patients had a coexisting systemic illness. None of the patients had a predisposing disease such as Gorlin's syndrome or xeroderma pigmentosum. Twenty-two (6.8%) patients had skin cancer in their family history. One hundred and twenty patients (37.5%) were smokers for more than ten years. The Fitzpatrick classifications of the patients were type 1 in 9 (2.8%) patients, type 2 in 129 (40.3%) patients, type 3 in 165 (51.6%) patients, and type 4 in 17 (5.3%) patients, respectively. There was no significant difference in the lesions that was related to a patient's skin type, in regard to subunits (Table 1).

Tumors most commonly occurred on the nose, with 107 (32.3%), followed by the orbital at 63 (19.1%), cheek 60 (18.1%), frontotemporal 42 (12.7%), auricular 23 (6.9%), scalp 21 (6.4%), perioral 9 (2.7%), and the chin-neck 6 (1.8%) regions. The size of the tumors was analyzed in 3 groups; 185 (55.9%) tumors were smaller than 10 mm in diameter, 143 (43.2%) were between 10 mm and 30 mm, and 3 (0.9%) were larger than 30 mm. In the nose and cheek regions the lesions were under 10 mm and in scalp region lesions between 10 and 30 mm were higher in number (*P* < 0.05).

Reports of clinical evaluation showed that all of the examined primary BCCs were of the nodular type and were either ulcerated or pigmented. With confirmation to histopathological analyses, 46 (13.8%) were of the mixed type (nodular + micronodular); 39 (11.7%) were of the micronodular type; 37 (11.2%) were of the superficial type; 31 (9.3%) were of the basosquamous type; 23 (6.9%) were of the adenoid type; 14 (4.2%) were of the sclerotic type of BCC. The rest of the lesions 141 (42.6%) were reported as being of the nodular type. Regarding the distribution of histopathologic patterns, no statistically significant difference was found in the entire head and neck region. In the scalp, frontotemporal, nose, and cheek regions, the nodular type tumor percentage was higher (*P* < 0.05). Two hundred and ninety-four excisions (88.9%) were undertaken under local anesthesia; 37 (11.1%) were done under general anesthesia.

The reconstruction method regarding the size of the defect was comprised of primary closure 84 (25.4%), full thickness skin grafting 54 (16.3%), and local flaps 192 (58%). One defect (0.3%) was reconstructed with the radial forearm free flap procedure (Table 2).

The mean follow-up time was at 33 ± 6.7 months. In nine lesions of the nine patients (2.7%) recurrence was observed. Recurrent lesions were in the scalp (*n* = 3), orbital (*n* = 2), and nose (*n* = 4) regions. The histopathological subtypes of these tumors were sclerotic (*n* = 4), micronodular (*n* = 3), mixed (*n* = 1), and nodular (*n* = 1). The recurrence time was between 6 months and 41 months (Table 3).

## 4. Discussion

BCC is the most common type of skin cancer in Caucasians, which predominantly occurs on the exposed parts of the body, with 75–85% of the lesions found in the head and neck regions [1, 2, 6–9]. According to our findings, more than half of the lesions are found in the nose (32.3%), orbital (19.1%), and cheek (18.1%) areas which are the most central and prominent parts of the entire head and neck region. These regions are also more prone to chronic sunlight exposure [2, 5, 10, 11].

Most of the BCC lesions are reported in the 40–79 age group, with the mean age of 62. In tropical regions and in patients with family history, BCC may occur in younger patients [2, 12]. We have had a statistically significant increase in the 60–70 age group (34.7%), in all the head and neck regions. But in other Mediterranean countries, the predominance is in the 70–80 age bracket [8, 13–15]. Regarding the patient's age and the location of the lesions, no significant difference was found. This can be explained with the siesta culture in these countries, which prevents their population from rush-hour sun exposure.

Men generally have up to 2 times higher rate of BCC [9, 10, 16]. In our study, when the whole head and neck region is encountered, there was no statistically significant difference between men (55%) and women (45%) that was consistent with other studies [10, 13, 17]. Most of our patients are from central Anatolia, where the population is generally comprised of men and women who both work during the daytime as farmers. When the subunits were evaluated, scalp, frontotemporal, and auricular lesions are more common in men, which can be explained by androgenic alopecia or short hair on men, when compared to the long hair or kerchief worn by women. Perioral lesions are found less in men, especially on the upper lip, which can be contributed to moustache on men. These findings are consistent with the report by Bastiaens et al. [18].

Phenotypic characteristics, such as fair skin type, red hair, and freckling are risk factors for BCC [9, 19]. Most of our patients were of type 2 (39.4%) and type 3 (51.6%), regarding the Fitzpatrick classification. This finding is similar to the literature [2, 10]. When the subunits were encountered, there was no significant difference in the lesions based on the patients' skin type.

Most of the lesions were found to be less than 10 mm in size (55.9%), when the whole head and neck region was evaluated. This finding is similar to other reports from Turkey [2]. This might be related to the recent increase in the public cognition of health issues and admission numbers at health facilities in Turkey. The ratio of tumors less than 10 mm was found to be higher in cosmetically more recognized regions, such as the nose and cheek (65.4% and 68.3%, resp.). This may be related to the social recognizability of the lesions.

Although a universally accepted classification scheme is lacking, commonly accepted clinical types are nodular, superficial, fibroepitelial, and morpheaform [9, 12]. Combinations of the latter three types with nodular BCC may occur. There are also histopathologic patterns, such as nodular, micronodular, adenoid, superficial, and sclerotic, which are referred to as subtypes of clinical type of nodular BCC [9, 20]. All of our patients were clinically evaluated as the nodular type BCC; histopathologically most of the lesions were nodular (42.2%). In the scalp, frontotemporal, nose, and cheek regions, a higher percentage of nodular type BCC was found.

The main goal of the BCC treatment is to eradicate the tumor with the safest and most cost-effective method available and to provide an aesthetically and functionally pleasant outcome. Even though different treatment modalities for BCC have been described, surgical excision is the most commonly preferred method for tumor removal [9].

In the literature, the 3 mm peripheral surgical margin is adequate for the clearance of 85% of small and well-defined BCCs, and the 4-5 mm margin will raise this to 95% [5]. It has been shown that loupe magnification can assist in reducing incomplete excision rates [20–22]. In our practice, with loupe magnification, the 3 to 10 mm excision margin was used, depending on the clinical features, size, and location of the lesion.

It has been shown that the recurrence rate for primary BCCs after surgical excision varies between 5% and 14% [1, 5]. Lesions in the head and neck region are at more risk for recurrence, when compared to lesions in trunk and extremities [1, 5, 7, 23]. Even though some factors, like anatomical localization, histopathological characteristics, and initial treatment strategy, have been proposed, there is lack of accepted understanding in the recurrence of the lesions [6]. Incomplete excision was reported as one of the risk factors for recurrence [6, 10, 20, 24]. The main difference of our study when compared with the previous studies is the precise evaluation of the factors effective on recurrence in completely excised BCCs. In our series, the overall recurrence rate was 2.7%. Recurrent lesions were in the scalp (*n* = 3), orbital (*n* = 2), and nose (*n* = 4) regions. Interestingly, all recurrences were in the median parts of head and neck region; this may be attributed to high recurrence rates in embryonic fusion planes [9, 25–27]. Histopathological subtype of recurrent tumors was mainly micronodular and sclerotic, which is more difficult to eradicate and has high risk of recurrence [28].

Our study revealed that recurrence is related to the localization and histopathologic subtype, whereas they were not related to age, sex, and size of the lesion. The low recurrence rates in our series might be due to the relatively low rates of histopathologically aggressive subtypes, excision with appropriate margin, and no positive surgical margins after surgery.

## 5. Conclusion

In conclusion, this study presents a relatively large number of series of surgically treated BCCs in the head and neck region. We would like to emphasize the importance of the preoperative evaluation of the patient keeping the epidemiology in mind, defining the surgical margins in order to get lower recurrence rates, and by motivating the patient for follow-up visits, in order to evaluate outcomes and diagnose recurrences.

## Figures and Tables

**Table 1:** Demographic analysis of patients.

	Total	Males	Females
Gender (%)	320	176 (55)	144 (45)
Age			
Mean	67.3	66.7	69.4
SD	12.76	12.11	13.32
Median	67	64	69
Range	18–95	28–90	18–95
Fitzpatrick skin type (%)			
1	9 (2.8)	3 (0.9)	6 (1.9)
2	129 (40.3)	69 (21.6)	60 (18.7)
3	165 (51.6)	93 (29.1)	72 (22.5)
4	17 (5.3)	11 (3.4)	6 (1.9)
Sun exposure history (%)	199 (62.2)	121 (37.8)	78 (24.4)
Systemic illness (%)	130 (40.6)	63 (19.7)	67 (20.9)
Family history (%)	22 (6.8)	8 (2.5)	14 (4.3)
Smoking history (%)	120 (37.5)	91 (28.4)	29 (9.1)

**Table 2:** Systematic analysis of patients' data with comparison to head and neck subunits.

Regions (Patients/Tumors)	Age					Sex		Size (mm)			Histopathological subtype							Anesthesia		Method of reconstruction			
	<50	50–60	60–70	70–80	80<	M	F	<10	10–30	<30	Nod	Mix	Mic	Sp	Bs	Ade	Sc	L	G	P	G	LF	DF
Scalp (21/21)	5	2	10	2	2	15	6	5	15	1	10	1	2	3	2	2	1	20	1	7	6	8	—
Frontotemporal (37/42)	5	4	17	12	4	31	11	20	21	1	14	3	6	7	3	4	5	34	8	15	10	16	1
Orbital (60/63)	6	11	21	19	6	30	33	32	31	—	25	7	9	5	10	5	2	51	12	16	13	34	—
Nose (104/107)	4	25	31	27	20	53	54	70	37	—	48	19	14	7	6	9	4	102	5	15	17	75	—
Cheek (60/60)	8	6	20	15	11	34	26	41	18	1	34	4	8	5	9	—	—	56	4	21	2	37	—
Auricula (23/23)	0	2	9	7	5	20	3	10	13	—	4	12		5	—	—	2	18	5	7	5	11	—
Perioral (9/9)	1	0	5	3	0	2	7	4	5	—	5	—		1	—	3	—	8	1	2	—	7	—
Chin-neck (6/6)	0	1	2	1	2	3	3	3	3	—	1	—		4	1	—	—	5	1	1	1	4	—

Total (320/331)	29	51	115	86	50	188	143	185	143	3	141	46	39	37	31	23	14	294	37	84	54	192	1

Nod indicates nodular; Mix: mixed; Mic: micronodular; Sp: superficial; Bs: basosquamous; Ade: adenoid; Sc: sclerotic; L: local; G: general; P: primary repair; G: full thickness skin graft; LF: local flap; and DF: distant flap.

**Table 3:** Clinical features of recurrent BCC.

	Regions	Specific localization	Age	Sex	Size (mm)	Histopathologic tumor type	Recurrence time	Treatment
1	Scalp	Left parietal	64	M	10–30	Nodular	36 months	Total excision
2	Scalp	Left parietal	66	F	<10	Sclerotic	25 months	Total excision
3	Scalp	Right parietal	62	M	10–30	Micronodular	41 months	Total excision
4	Orbital	Left medial cantus	63	M	10–30	Sclerotic	8 months	Total excision
5	Orbital	Right medial cantus	73	F	10–30	Sclerotic	6 months	Total excision
6	Nose	Dorsum	68	M	<10	Micronodular	18 months	Total excision
7	Nose	Right alar region	73	F	<10	Sclerotic	20 months	Total excision
8	Nose	Nose tip	71	F	<10	Mixed	22 months	Total excision
9	Nose	Left alar region	79	M	<10	Micronodular	9 months	Total excision
